# SKN-1 and Nrf2 couples proline catabolism with lipid metabolism during nutrient deprivation

**DOI:** 10.1038/ncomms6048

**Published:** 2014-10-06

**Authors:** Shanshan Pang, Dana A. Lynn, Jacqueline Y. Lo, Jennifer Paek, Sean P. Curran

**Affiliations:** 1 Davis School of Gerontology, University of Southern California, 3715 McClintock Avenue, Los Angeles, California 90089, USA; 2 Department of Molecular and Computational Biology, Dornsife College of Letters, Arts, and Sciences, University of Southern California, Los Angeles, California 90089, USA; 3 Department of Biochemistry and Molecular Biology, Keck School of Medicine, University of Southern California, Los Angeles, California 90089, USA

## Abstract

Mechanisms that coordinate different metabolic pathways, such as glucose and lipid, have been recognized. However, a potential interaction between amino acid and lipid metabolism remains largely elusive. Here we show that during starvation of *Caenorhabditis elegans*, proline catabolism is coupled with lipid metabolism by SKN-1. Mutation of *alh-6*, a conserved proline catabolic enzyme, accelerates fat mobilization, enhances the expression of genes involved in fatty acid oxidation and reduces survival in response to fasting. This metabolic coordination is mediated by the activation of the transcription factor SKN-1/Nrf2, possibly due to the accumulation of the *alh-6* substrate P5C, and also requires the transcriptional co-regulator MDT-15. Constitutive activation of SKN-1 induces a similar transcriptional response, which protects animals from fat accumulation when fed a high carbohydrate diet. In human cells, an orthologous *alh-6* enzyme, ALDH4A1, is also linked to the activity of Nrf2, the human orthologue of SKN-1, and regulates the expression of lipid metabolic genes. Our findings identify a link between proline catabolism and lipid metabolism, and uncover a physiological role for SKN-1 in metabolism.

## Abstract (web summary)

The coordinated metabolism of cellular nutrients is important to maintain energy homeostasis, particularly if nutrients are scarce. Here, the authors report that the sensor protein SKN-1/Nrf2 links catabolism of the amino acid proline with lipid metabolism in *C. elegans* and in cultured human cells.

## Graphical abstract

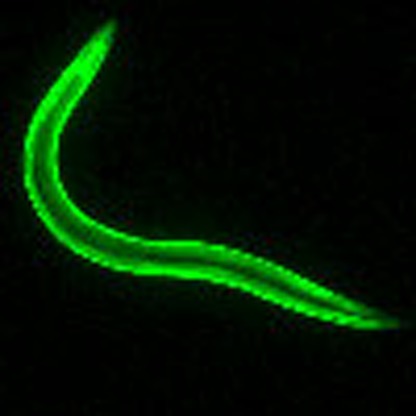

Animals maintain energy homeostasis through the coordinated metabolism of available intracellular nutrients, including glucose, lipids and amino acids. To do so, animals employ complex but elegant molecular mechanisms to integrate the metabolism of these nutrients. In mice for example, under well-fed conditions, the liver X receptor integrates hepatic glucose metabolism and lipogenesis by acting as a glucose sensor^1^. Glucose-mediated ChREBP activation in adipose tissue activates fatty acid synthesis, thus connecting glucose and lipid metabolism^2^. Although mechanisms such as these linking glucose and lipid metabolism have been well recognized, it remains largely elusive whether and how the metabolism of amino acids and lipids, two major nutrients for fasting responses, are coordinated.

During periods of nutrient deprivation, stored lipids and amino acids are used instead of dietary glucose to satisfy organismal energy requirements. Lipids are mobilized as an energy resource through lipolysis and fatty acid oxidation (FAO). Meanwhile, amino acids, another important energy resource, can either be directly oxidized or converted to glucose, and then oxidized by organs with an obligatory glucose requirement^3^. Based on the universal importance of these metabolic pathways during starvation, it is possible that their metabolism may be coupled together during fasting, and that this link would be well conserved.

SKN-1 is the worm homologue of the mammalian transcription factor Nrf2, both of which share a conserved cytoprotective function in the response to cellular electrophiles^4^. In addition, SKN-1 is a well-known longevity factor that is activated in many long-lived mutant backgrounds with altered metabolic homeostasis and is indispensable for the lifespan extension of those mutants^5^,^6^,^7^,^8^. Recently, we reported that gain-of-function mutations in *skn-1* lead to a starvation-like status in *C. elegans* and induce the expression of several metabolic genes^9^; however, the full extent to which SKN-1 participates in organismal physiology and metabolism remains unknown.

*C. elegans* is an established model for studying conserved pathways that govern lipid metabolism^10^,^11^,^12^,^13^. In this study, by using a *C. elegans* strain with a mutation in a conserved proline catabolic gene, we investigate the role of proline catabolism in the organismal response to fasting and discover that proline catabolism is coupled with fasting lipid utilization by the transcription factor SKN-1/Nrf2.

## Results

### Loss of *alh-6* accelerates lipid mobilization during fasting

**Figure 1:**
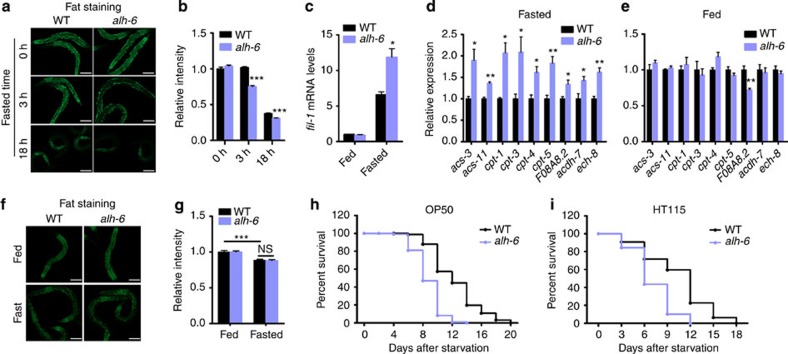
Mutation of *alh-6* enhances fat mobilization and the expression of FAO genes during starvation. (**a**,**b**) Nile Red staining of OP50 fed wild-type and *alh-6* mutants in response to fasting. The representative images are shown in **a** (scale bar, 100 μm) and quantitative data are shown in **b** (*n*=12 for 0 h of wild-type and *alh-6 (lax105)* mutants, *n*=13 for 3 h of *alh-6* mutants, *n*=9 for other groups). (**c**) Expression of *fil-1* in response to 3 h fasting (*n*=3). (**d**,**e**) Expression of FAO genes under 3 h fasted (**d**) and well-fed (**e**) conditions (*n*=3). (**f**,**g**) Nile Red staining of wild type and *alh-6* mutants fed HT115 in response to 3 h fasting. The representative images are shown in **f** (scale bar, 100 μm) and quantitative data are shown in **g** (*n*=8 for fed wild type, *n*=9 for fed *alh-6* mutants and fasted wild type, *n*=10 for fasted *alh-6* mutants). (**h**,**i**) Survival rate of wild type and *alh-6* mutant worms during starvation when fed OP50 (**h**) or HT115 (**i**) diet before starvation. Data were presented as mean±s.e.m. (*P<0.05, **P<0.01, ***P<0.001, Student’s *t*-test, versus wild-type controls under same treatment unless specifically indicated).

In a previous study, we identified mutations in the *C. elegans* gene *alh-6*, an evolutionarily conserved mitochondrial enzyme involved in the catabolism of proline (Supplementary Fig. 1a)^14^. In this study, we asked whether mutation of *alh-6* would affect lipid homeostasis. We first compared the fat content between wild-type and *alh-6* mutant worms. When fed the standard *Escherichia coli* OP50 diet *ad libitum*, wild-type and *alh-6* mutant worms store similar levels of intestinal fat as measured by Nile Red staining (Fig. 1a,b) or Oil Red O staining (Supplementary Fig. 1b). However, within a short 3 h exposure to starvation, *alh-6* mutants rapidly mobilized intestinal lipids as compared with wild-type worms, which had yet to measurably use these nutrient stores (Fig. 1a,b and Supplementary Fig. 1b). Following a 18 h long-term starvation period, *alh-6* mutants continued the hypermobilization of intestinal fat when compared with wild-type animals, which at this time point had also significantly depleted stored lipids (Fig. 1a,b). Thus, *alh-6* mutants further enhance the mobilization of stored fat in response to food deprivation. This data indicates that *alh-6* regulates lipid mobilization during starvation and implies that proline catabolism is coupled with lipid metabolism in response to nutrient depletion.

We next examined the expression of genes involved in lipid metabolism. The expression levels of *pod-2/ACC1* and *fasn-1/FASN*, the key enzymes in fatty acid synthesis, were comparably inhibited in wild-type and *alh-6* mutant worms in response to fasting (Supplementary Fig. 1c). However, the expression of the fasting-induced lipase-1 (*fil-1*), a key lipolytic enzyme responsible for the *C. elegans* starvation response^15^, was significantly induced in *alh-6* mutants during starvation above the measured increase in starved wild-type animals (Fig. 1c). Under fasted conditions, fat is used through mitochondrial and peroxisomal FAO^16^,^17^,^18^,^19^. We tested the expression of all annotated FAO enzymes in animals starved for 3 h (Supplementary Tables 1 and 2)^20^. Consistent with the enhanced fat mobilization, *alh-6* mutants exhibited increased expression of several FAO enzymes, specifically under fasted (Fig. 1d and Supplementary Table 1) but not well-fed conditions (Fig. 1e). These enzymes constitute several main steps of mitochondrial and peroxisomal FAO pathways^20^. Thus, fasted *alh-6* mutants enhance lipid mobilization characterized by increased expression of genes involved in lipolysis and FAO but not *de novo* lipogenesis.

We identified several *alh-6*-sensitive FAO enzymes that were upregulated to enhance the wild-type fasting response (Supplementary Fig. 1d). In addition, we also discovered an increase in the expression of a subset of FAO genes that results in the derepression of targets that are inhibited in starved wild-type animals (Supplementary Fig. 1e)^20^. These data indicate that not all FAO enzymes are universally used under all starvation conditions, but rather some may specifically respond to the fasted state in the context of the *alh-6* mutant background.

As mutations in *alh-6* cause premature ageing in a diet-dependent manner^14^, we asked whether the enhanced lipid mobilization phenotype identified above was also dependent on diet. Remarkably, the rapid depletion of intestinal lipid stores was abrogated when *alh-6* mutants were raised on another common *C. elegans* diet, the *E. coli* K-12 strain HT115. Specifically, on this dietary regimen, *alh-6* mutants exhibited comparable levels of fat mobilization in response to fasting (Fig. 1f,g) and showed no significant changes in the expression of FAO genes (Supplementary Fig. 1f) when compared with wild-type controls. Thus, the diet ingested before starvation establishes an organism’s metabolic adaptation programme during food deprivation.

We also asked whether mutation of *alh-6* affected animal survival during fasting. We found that *alh-6* mutants display significantly reduced animal survival in response to starvation (Fig. 1h and Supplementary Table 3), further indicating that *alh-6* is an important regulator of fasting adaptation. Intriguingly, the reduced survival of *alh-6* mutant worms during fasting is not dependent on the diet before starvation (Fig. 1i and Supplementary Table 3), suggesting the role of *alh-6* for survival during acute and long-term fasting are different.

### SKN-1 mediates lipid metabolism responses in *alh-6* mutants

**Figure 2:**
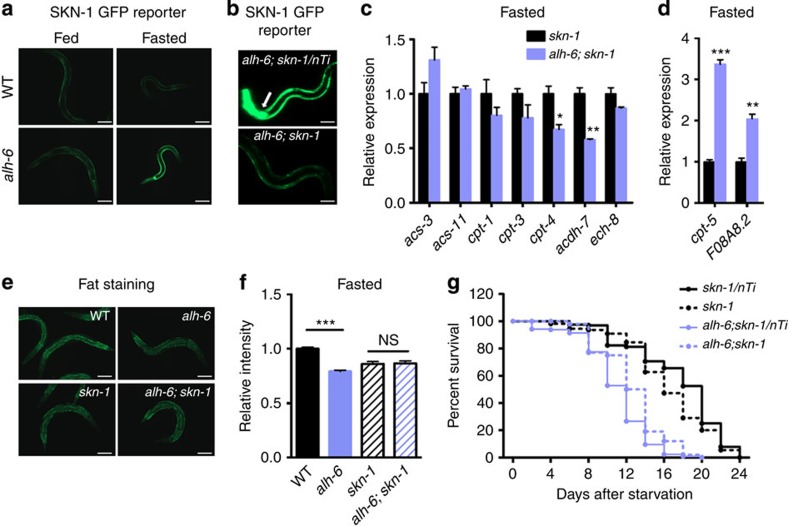
SKN-1 coordinates proline and lipid metabolism during starvation of *C. elegans.* (**a**) Mutation of *alh-6* activates *gst-4p*::GFP, a SKN-1 transcriptional activity reporter, during overnight fasting. Scale bar, 100 μm. (**b**) Mutation of *skn-1* abolished the activation of *gst-4p*::GFP. The presence of the *skn-1* balancer is indicated by green fluorescent protein expression in the pharynx as pointed out by the arrow. Scale bar, 100 μm. (**c**,**d**) The increased expression of FAO genes in 3 h fasted *alh-6* mutant is either dependent (**c**) or independent (**d**) on *skn-1* (*n*=3). (**e**,**f**) Nile Red staining of worms with indicated genotypes under 3 h fasted condition. The representative images are shown in **e** (scale bar, 100 μm) and quantitative data are shown in **f** (*n*=11 for wild type, *n*=10 for other groups). (**g**) Effect of *skn-1* mutation on the starvation survival rate of *alh-6* mutant worms. Data were presented as mean±s.e.m. (*P<0.05, **P<0.01, ***P<0.001, Student’s *t*-test versus controls under same treatment unless specifically indicated).

The increased expression of FAO genes in fasted *alh-6* mutants indicates the existence of a transcriptional response that monitors and responds to perturbations in cellular proline metabolism. A role for the transcription factor SKN-1 has been documented under conditions of oxidative stress^4^ and lifespan extension^5^,^6^,^7^,^8^, where nutrient availability is either perceived as reduced or is actually reduced. Furthermore, we recently found that gain-of-function mutations in *skn-1* induce a starvation-like state^9^. As such, we proposed that a SKN-1-mediated transcriptional programme could mechanistically link proline and fatty acid metabolism. Under well-fed conditions, the expression of the SKN-1 transcriptional activity reporter *gst-4p*::GFP was similar between juvenile wild-type and *alh-6* mutant worms (Fig. 2a). However, when starved, the SKN-1 reporter was dramatically activated in *alh-6* mutants but not in wild-type controls (Fig. 2a). Furthermore, loss of SKN-1 function through null mutation substantially reduced the fasting-dependent activation of the SKN-1 reporter in *alh-6* mutants (Fig. 2b). We conclude that *alh-6* mutants activate SKN-1 during food deprivation.

We next asked whether SKN-1 mediated the enhanced mobilization of stored lipids in fasted *alh-6* mutants. We found that seven out of nine FAO genes with increased expression in the fasted *alh-6* mutants were no longer upregulated in the absence of SKN-1 (Fig. 2c). The expression of two FAO genes were still activated independently of SKN-1 in the *alh-6* mutants during fasting (Fig. 2d), indicating the existence of other compensatory pathway(s) that function in parallel to SKN-1. Most importantly, a loss-of-function mutation in *skn-1* abrogated the enhanced depletion of intestinal lipid stores observed in *alh-6* mutant worms after fasting (Fig. 2e,f), indicating an essential role for SKN-1 in mediating this fasting metabolic response. However, mutation of *skn-1* could not significantly reverse the reduced starvation survival rate of *alh-6* mutant worms (Fig. 2g), further indicating the mechanistic differences between the lipid metabolism and survival responses in fasted *alh-6* mutants.

We previously reported that *alh-6* mutations were capable of activating the SKN-1 reporter under fed conditions, but only after day 3 of the adult reproductive period^14^. Despite activation of SKN-1 at this time point in adult life, these *alh-6* mutants did not induce a similar transcriptional change in FAO genes (Supplementary Fig. 2a) and do not reduce levels of stored fat (Supplementary Fig. 2b). These findings indicate a phenotypic difference between the same SKN-1-inducing mutation under different physiologic contexts, which suggests that the SKN-1-mediated lipid response represents a specific metabolic response to the *alh-6* mutation during starvation, and not merely an indirect side effect of global SKN-1 activation.

In our previous study, we also reported that accumulation of the *alh-6* substrate P5C and the subsequent generation of mitochondrial reactive oxygen species (ROS), such as hydrogen peroxide, are responsible for the premature ageing phenotype observed in adult *alh-6* mutants^14^. Treatment with the antioxidant N-acetylcysteine (NAC) completely abrogated the shortened lifespan of *alh-6* mutants. As such, we evaluated a role for mitochondrial ROS in *alh-6*-mediated fasting lipid responses following NAC treatment. We found that although NAC treatment blocked the SKN-1 reporter activation induced by exposure to arsenite, an inducer of oxidative stress, NAC had no effect on the SKN-1 activation observed in fasted *alh-6* mutants (Supplementary Fig. 2c). Moreover, NAC-treated *alh-6* mutant worms still exhibited accelerated fat mobilization in response to fasting (Supplementary Fig. 2d). These data suggest that mitochondrial oxidative stress is not involved in SKN-1 activation and the enhanced lipid metabolism observed in fasted *alh-6* worms. As SKN-1 can respond to multiple types of cellular stress, it is possible that additional, non-oxidative stress signals caused by P5C accumulation are responsible for the observed SKN-1 activation and lipid changes.

### SKN-1 protects against diet-induced fat accumulation

**Figure 3:**
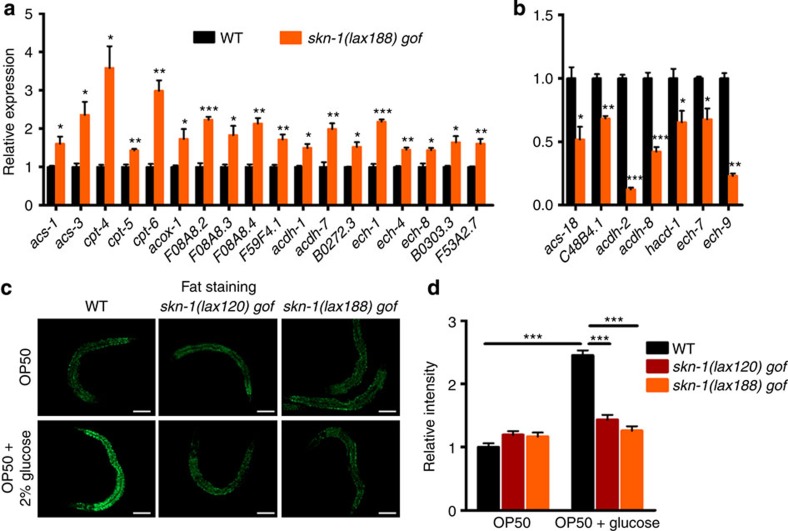
Constitutive activation of SKN-1 protects animals from HCD-induced fat accumulation. (**a**,**b**) Expression of FAO genes that are upregulated (**a**) or downregulated (**b**) by *skn-1(lax188)* gain-of-function (gof) mutation (*n*=3). (**c**,**d**) Nile Red staining of wild type, *skn-1(lax120)* and *skn-1(lax188)* gain-of-function mutants fed OP50 or OP50 plus 2% glucose. The representative images are shown in **c** (scale bar, 100 μm) and quantitative data are shown in **d** (*n*=5 for wild type fed OP50, *n*=10 for wild type fed OP50 plus 2% glucose, *n*=8 for *skn-1 (lax188)* fed OP50, *n*=7 for other groups). Data were presented as mean±s.e.m. (*P<0.05, **P<0.01, ***P<0.001, Student’s *t*-test versus controls under same treatment unless specifically indicated).

Subsequently, we tested whether *skn-1* gain-of-function mutations could induce a similar transcriptional response, and more importantly, if they result in a change in stored lipids. We discovered that well-fed *skn-1* gain-of-function mutant worms upregulated a large number of FAO genes (Fig. 3a), which is consistent with our previous observation that *ad libitum*-fed *skn-1* gain-of-function animals behave as if they are starved^9^. Intriguingly, some FAO genes were found to be downregulated in *skn-1* gain-of-function mutants as compared with wild-type controls (Fig. 3b), further supporting the idea that unique FAO enzymes are differentially mobilized in response to particular metabolic stresses. Although, there was a larger set of lipid metabolism genes altered in the *skn*-1 gain-of-function mutants, there was a clear overlap with the genes increased in the *alh-6* mutants during fasting (Supplementary Fig. 3a). This gene expression pattern indicates a SKN-1-dependent pathway for inducing an organism-level metabolic response that is defined by the activation of fatty acid utilization pathways in both the *skn-1* gain-of-function mutants and SKN-1-activating *alh-6* mutants under conditions of fasting. We then measured the fat content of those gain-of-function mutant worms. Although transcriptionally poised for increased oxidation of stored fat, well-fed *skn-1* gain-of-function mutant animals exhibited relatively similar levels of fat content compared with well-fed wild-type controls as measured by Nile Red staining (Fig. 3c,d), and a minor decrease of fat as revealed by Oil Red O staining (Supplementary Fig. 3b). We hypothesized that the induction of FAO enzymatic activity in mutants with constitutive SKN-1 activation might only significantly impact lipid homeostasis, at the organismal level, under conditions of metabolic stress. We thus examined the function of constitutively activated SKN-1 in animals fed a high carbohydrate diet (HCD)^21^, which serves as model that mimics the diet-induced obesity observed in mammals. We found that addition of 2% glucose to the standard diet could significantly induce a 250% increase in stored intestinal fat in wild-type *C. elegans*, as compared with worms feeding on a normal diet (Fig. 3c,d and Supplementary Fig. 3b). Strikingly, when *skn-1* gain-of-function mutants were fed the HCD, they did not manifest this increased lipid phenotype (Fig. 3c,d and Supplementary Fig. 3b). These data suggest that constitutive SKN-1 activation can transcriptionally predispose animals to successfully cope with dietary insults, and that this adaptive capacity is capable of suppressing the lipid accumulation phenotype resulting from a HCD.

### Aldh4a1 and Nrf2 regulate FAO genes in human cells

**Figure 4:**
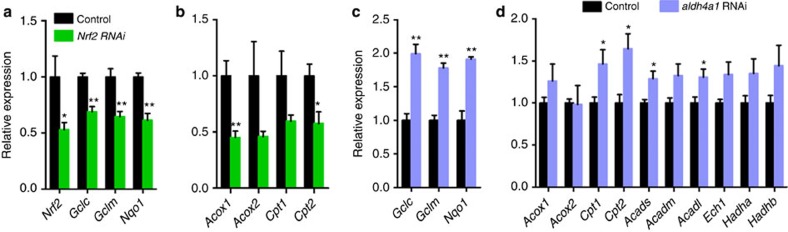
Conserved regulation of Nrf2 activity and FAO genes by Aldh4a1. (**a**,**b**) Knockdown of *Nrf2* inhibits expression of its canonical target genes (**a**) and FAO genes (**b**) (*n*=3 for control, *n*=5 for *Nrf2* RNAi). (**c**,**d**) Knockdown of *aldh4a1* induces expression of Nrf2 target genes (**c**) (*n*=3) and FAO genes (**d**) (*n*=6). Data were presented as mean±s.e.m. (*P<0.05, **P<0.01, Student’s *t*-test versus controls).

We next examined the possible conservation of the *alh-6*/*skn-1* pathway in human cells. We first asked whether Nrf2, the human orthologue of SKN-1, also regulated the expression of FAO genes in human cells. Although Nrf2 activity has been linked to cancer cell metabolism and lipid biosynthesis in rodents^22^, its role in regulating FAO has not been established. We found that RNA interference (RNAi)-mediated knockdown of *Nrf2* inhibited the expression of canonical Nrf2 target genes (Fig. 4a) and also several FAO genes in 293T cells (Fig. 4b), indicating that Nrf2 is a regulator of FAO genes in human cells. Next, we performed small interfering RNA (siRNA) knockdown of *aldh4a1*, the human orthologue of worm *alh-6*, and examined the effects on gene expression. Remarkably, *aldh4a1* RNAi not only induced the expression of Nrf2 targets (Fig. 4c), which is indicative of Nrf2 activation, but also induced the expression of a subset of FAO genes (Fig. 4d). These data implicate that the SKN-1/Nrf2-mediated regulatory axis between proline and lipid metabolism has functional conservation from invertebrates to humans.

### MDT-15 co-regulates lipid metabolism with SKN-1

**Figure 5:**
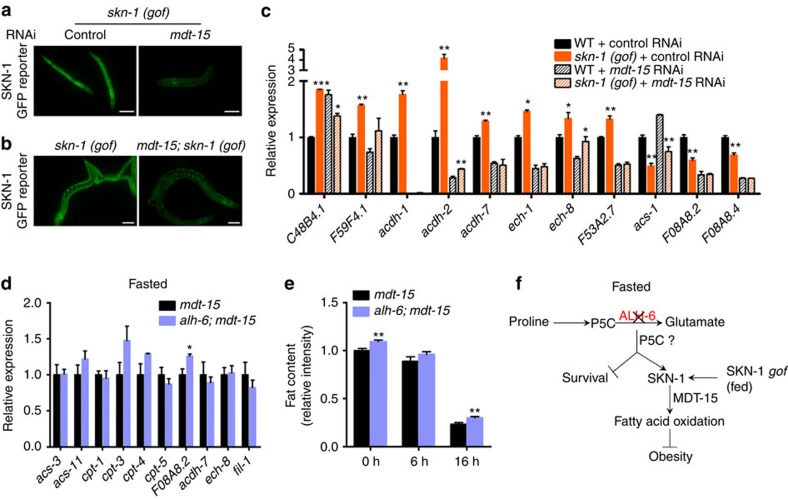
MDT-15 is a co-factor for SKN-1-mediated lipid metabolism. (**a**,**b**) RNAi mediated knockdown (**a**) or point mutation (**b**) of *mdt-15* abolishes the activation of *gst-4p*::GFP in *skn-1* gain-of-function (gof) mutants *skn-1 (lax188)*. Scale bar, 100 μm. (**c**) Expression of FAO genes that are regulated by *skn-1 (gof)*-fed HT115 bacteria containing L4440 control or *mdt-15* RNAi plasmids (*n*=2 for *skn-1 (lax188)*-fed control RNAi, *n*=3 for other groups). (**d**) The expression of *alh-6*-mediated FAO genes is largely dependent on *mdt-15* (*n*=3). (**e**) Fat content of *mdt-15* and *alh-6; mdt-15* mutants during starvation as measured by Nile Red staining (*n*=13 for 0 h of *mdt-15* and 16 h of *alh-6; mdt-15* mutants, *n*=10 for 6 h of *mdt-15* and *alh-6; mdt-15* mutants, *n*=11 for 16 h of *mdt-15* mutants, *n*=12 for 0 h of *alh-6; mdt-15* mutants). (**f**) Model: during fasting, mutation of the proline catabolic gene *alh-6* activates SKN-1, possibly through accumulation of metabolic intermediate P5C to mediate transcriptional programme for the induction of FAO genes, which also requires co-regulator MDT-15. Constitutively activated SKN-1 induces similar transcriptional changes in FAO genes that protect animals from diet-induced obesity. Data were presented as mean±s.e.m. (*P<0.05, **P<0.01, ***P<0.001, Student’s *t*-test versus controls under same treatment unless specifically indicated).

In light of the multitude of responses that are influenced by SKN-1/Nrf2, we predicted that the SKN-1/Nrf2 lipid metabolism response we identified would require additional transcriptional co-regulators. To identify possible co-regulators of SKN-1 in modulating lipid metabolism, we first screened an RNAi library targeting all annotated transcriptional regulators and DNA-binding proteins in *C. elegans*, looking for suppression of the SKN-1 reporter activation observed in the *skn-1* gain-of-function mutants^9^. We discovered that *mdt-15* was required for SKN-1 reporter activation, as RNAi targeting *mdt-15* significantly abolished the reporter activation (Fig. 5a). Moreover, in a complementary approach, we performed a classical ethyl methanesulfonate (EMS) mutagenesis screen for suppressors of the SKN-1 reporter activation in the *skn*-1 gain-of-function mutant background. We isolated a single complementation group that mapped to the centre of LGIII and identified a Gly to Glu mutation in MDT-15 (Fig. 5b and Supplementary Fig. 4). MDT-15 is a transcriptional regulator of lipid metabolism^23^ and has been found to physically interact with SKN-1 (ref. 24). We then subsequently tested the role for MDT-15 in SKN-1-mediated lipid metabolism by examining the effect of *mdt-15* RNAi on lipid gene expression in the *skn-1* gain-of-function mutants. These mutants also display enhanced expression of FAO genes when raised on the control RNAi bacteria HT115 (Fig. 5c). However, it is notable that the gene expression changes observed are not identical to those when animals were fed the OP50 *E. coli* B diet (Fig. 3a,b), further supporting the diet-dependent response of SKN-1 function in lipid metabolism. RNAi knockdown of *mdt-15* largely abolished the effects of *skn-1* gain-of-function mutation on FAO gene expression (Fig. 5c), suggesting MDT-15 is a critical cofactor for the transcription of these targets. Moreover, in the *mdt-15* mutant background, *alh-6* mutant animals no longer exhibited the increased expression of FAO genes (Fig. 5d) or enhanced fat mobilization in response to fasting (Fig. 5e). Together, our results refine the molecular mechanisms by which SKN-1 and MDT-15 cooperate to maintain lipid homeostasis and define MDT-15 as a co-regulator of SKN-1-dependent lipid metabolism.

## Discussion

In this study, we reveal a novel link between proline and lipid metabolism, and identify a SKN-1/Nrf2-dependent mechanism that coordinates these two metabolic pathways (Fig. 5f). How does mutation of *alh-6* lead to SKN-1 activation and lipid responses during fasting? A possible mechanism is that accumulation of the *alh-6* substrate P5C induces SKN-1 activation and fat mobilization. A recent study in mammalian adipose cells has reported that during nutrient withdrawal, the activation of *prodh*, the enzyme producing P5C, can induce lipase expression^25^. This finding supports the model for P5C as a conserved metabolic signal in activating SKN-1 and regulating fat mobilization during starvation. Generation and utilization of animals with mutations in or reduced expression of *prodh*, the P5C generating enzyme, will be valuable for testing this theory. Although Barbato *et al*.^25^ identified a role for ROS, this could represent the differences between our experimental models and readouts: apoptosis and inflammation in 3T3 cells versus organismal lipid depletion, or the inherent differences in responses for specific tissues. A more thorough understanding of the coordination of such responses will be of significant interest for future studies.

SKN-1 is an essential transcription factor mediating cellular stress responses, such as oxidative stress and immune defense. Recent gene profile analyses indicate that SKN-1 may also be an important regulator of metabolism. In this study, we identify a physiological role for SKN-1 in metabolism, coordinating proline catabolism with lipid utilization during fasting. SKN-1 is thus a critical transcription factor that responds to diverse cellular stresses, including metabolic stress. Disruption of *alh-6*-dependent proline catabolism during fasting induces changes in the expression of several FAO genes, most of which are regulated in a SKN-1-dependent manner. Intriguingly, six of the seven SKN-1-dependent genes we identified contain three to six conserved SKN-1-binding sites in their 2 kb promoters (Supplementary Fig. 3c); a SKN-1-binding site is generally found by chance only once in the same length of the genome^4^,^26^,^27^. This data indicates that some of these FAO genes may be direct targets of SKN-1.

We find that a subset of FAO genes, whose expression is inhibited by starvation in wild-type animals, is derepressed in fasted *alh-6* mutant worms. This finding indicates physiological differences of the collection of FAO genes in the genome. We propose that *C. elegans* use unique FAO enzymes in response to distinct metabolic stress conditions: some metabolic enzymes can have overlapping functions and/or can be activated in response to specific cellular needs.

Another interesting finding of our study is that compromised *alh-6*-mediated proline catabolism regulates lipid metabolism during fasting in a diet-dependent manner. Although the response is triggered under a condition without food, our data suggests that dietary intake before food deprivation could predetermine an organism’s response during starvation. The different nutritional composition between the OP50 and HT115 diets may lead to preferential use of specific energy substrates. We propose that, when fasted, animals previously fed an OP50 diet may rely more on mitochondrial *alh-6* proline catabolism than those fed the HT115 diet. When *alh-6* is mutated, animals fed the OP50 diet might be more stressed when exogenous nutrients are no longer available. This condition thereby activates the lipid metabolism response through SKN-1 and MDT-15. Intriguingly, the diet consumed before fasting can have significant effects on mouse behaviour during food deprivation^28^, which suggests that dietary pre-determination of the adaptive response to starvation is also evolutionarily conserved.

Abnormal fat accumulation induced by diet underlies multiple metabolic diseases, such as obesity and type II diabetes. We and others show that a diet supplemented with high glucose can induce massive lipid accumulation in *C. elegans*, indicating the possibility of using this as a model to study diet-induced fat accumulation. In this study, we find that *skn-1* gain-of-function mutation protects animals against the increased lipid storage phenotype when fed a HCD. This finding implicates SKN-1 as a potential target for the treatment of abnormal lipid metabolism. Furthermore, Nrf2 can similarly regulate FAO genes in human cells revealing the evolutionary importance of this cellular metabolic response system. Thus, studies regarding the possible use of Nrf2 pathway agonists for regulating lipid metabolism and improving its related metabolic diseases will be of high clinical importance.

## Methods

### *C. elegans* growth conditions and strains

*C. elegans* were cultured using standard techniques at 20 °C^29^. The following strains were used: wild-type N2 Bristol, SPC207: *skn-1 (lax120)*, SPC227: *skn-1 (lax188)*, SPC321: *alh-6 (lax105)*, CL2166: *gst4-p::gfp*, SPC276: *skn-1 (lax188); mdt-15 (lax225); gst4-p::gfp*, VC1772: *skn-1 (ok2315) IV/nTi[qIs51] (IV; V)* and XA7702: *mdt-15 (tm2182)*. Double or triple mutants were generated by standard genetic techniques.

### Starvation assay

For starvation, synchronized L1 animals were added to nematode growth medium (NGM) plates seeded with indicated bacteria. After 2 days at 20 °C, L4 animals were collected, washed with M9 buffer at least three times and then subjected to fasting in M9 liquid with shaking for indicated time before collection for further analysis. Starvation survival assay was performed as previous described^30^. Briefly, gravid worms that did not experience starvation for at least two generations were used for egg preparation. After 24 h, synchronized L1 animals were resuspended in M9 at a concentration of two worms per microlitre. Starvation culture was mixed by constant rocking. Every 2 days, a portion of animals was recovered on normal OP50-seeded NGM plates. Animals that resumed development were considered to be surviving.

### Nile Red staining

Nile Red staining was performed as previously described^31^. Briefly, animals of indicated genotypes were collected, fixed in 40% isopropanol at room temperature for 3 min and stained in 3 μg ml^−1^ Nile Red working solution in dark for 2 h. Worms were then washed with M9 for at least 30 min, mounted on slides and imaged under the green fluorescent protein channel of microscope Zeiss Axio Imager with Zen software package. Fluorescent density was measured using ImageJ software. Approximately ten animals from each experiment (*n*) were used to calculate the fluorescent density.

### Oil Red O staining

Animals of indicated genotypes were collected and fixed in 1% formaldehyde in PBS for 10 min. Next, samples were frozen and thawed three times with dry ice/ethanol bath. Worms were washed with PBS three times before staining with freshly prepared Oil Red O working solution. Worms were stained while rotating for 30 min, washed again with PBS for 15 min, mounted on slides and imaged under a bright-field illumination.

### RNAi treatment

HT115 bacteria containing specific double stranded RNA-expression plasmids were seeded on NGM plates containing 5 mM isopropyl-β-D-thiogalactoside and 50 μg ml^−1^ carbenicillin. RNAi was induced at room temperature for 24 h^32^. Synchronized L1 animals were added to those plates to knockdown indicated genes.

### Quantitative reverse transcription–PCR

Quantitative reverse transcription–PCR was performed as previously described^14^. Briefly, worms of the indicated genotype and stages were collected, washed in M9 buffer and then homogenized in Trizol reagent (Life Technologies). RNA was extracted according to the manufacturer’s protocol. DNA contamination was digested with DNase I (New England Biolabs) and subsequently RNA was reverse-transcribed to complementary DNA by using the SuperScript III First-Strand Synthesis System (Life Technologies). Quantitative PCR was performed by using SYBR Green (BioRad). The expression levels of *snb-1* and *actin* were used to normalize samples in worms and human cells, respectively. Primer sequences listed in Supplementary Table 2.

### Human cell culture

293T cells were cultured in DMEM supplemented with 10% fetal bovine serum. At 50–70% confluence, cells were transfected with control, *Nrf2/Nfe2l2(s9492)* or *aldh4a1(s16484)* Silencer Select (Life Technologies) siRNA by using Lipofectamine RNAiMax (Life Technologies). After 24 h, cells were washed and collected in Trizol reagent (Invitrogen) for further RNA extraction. For examining the Nrf2 target genes in *aldh4a1* siRNA experiment, cells were collected 48 h after transfection.

### Statistical analysis

Data are presented as mean±s.e.m. Data were analysed by using unpaired Student’s *t*-test. *P*<0.05 was considered as significant.

## Author contributions

S.P. and S.P.C. designed the study; S.P., D.A.L., J.Y.L., J.P. and S.P.C. performed the experiments; S.P., D.A.L. and S.P.C. analysed data and wrote the manuscript.

## Additional information

**How to cite this article:** Pang, S. *et al*. SKN-1 and Nrf2 couples proline catabolism with lipid metabolism during nutrient deprivation. *Nat. Commun.* 5:5048 doi: 10.1038/ncomms6048 (2014).

## Supplementary Material

Supplementary Information Supplementary Figures 1-4 and Supplementary Tables 1-3
