# Supplementary material for: SKN-1 and Nrf2 couples proline catabolism with lipid metabolism during nutrient deprivation
Source: Nat Commun. 2014 Oct 6;5:5048. doi: 10.1038/ncomms6048 (PMC4205844; doi:10.1038/ncomms6048)
Supplement: Supplementary Information — Supplementary Figures 1-4 and Supplementary Tables 1-3 [file ncomms6048-s1.pdf]

Supplemental Figures

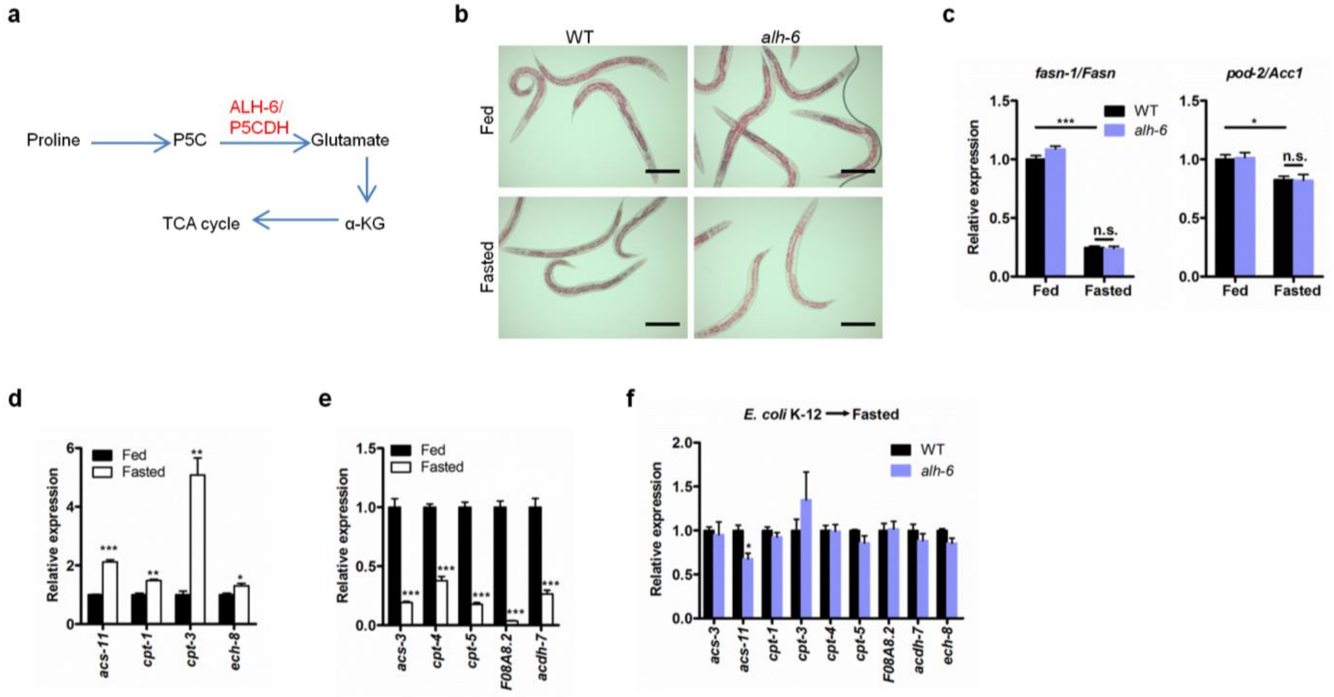

Supplementary Figure 1: Analysis of lipid metabolism in *alh-6* mutants during starvation. (a) Schematic of amino acid catabolism pathways regulated by ALH-6, which encodes the *C. elegans* 1-pyrroline-5-carboxylate dehydrogenase (P5CDH). (b) Oil Red O staining of OP50 fed wild type and *alh-6* mutants in response to three hours of fasting. Scale bar: 100um. (c) Expression of fatty acid synthesis genes under well-fed or three hours fasting conditions (n = 3). (d-e) *alh-6* dependent FAO genes are either upregulated (d) or downregulated (e) by starvation in wild type worms (n = 3). (f) Expression of FAO genes under three hours fasting conditions in worms fed HT115 bacteria (n = 3). Data are presented as mean ± SEM. (*p < 0.05, **p < 0.01, ***p < 0.001, student's t-test, versus controls under same treatment unless specifically indicated.)

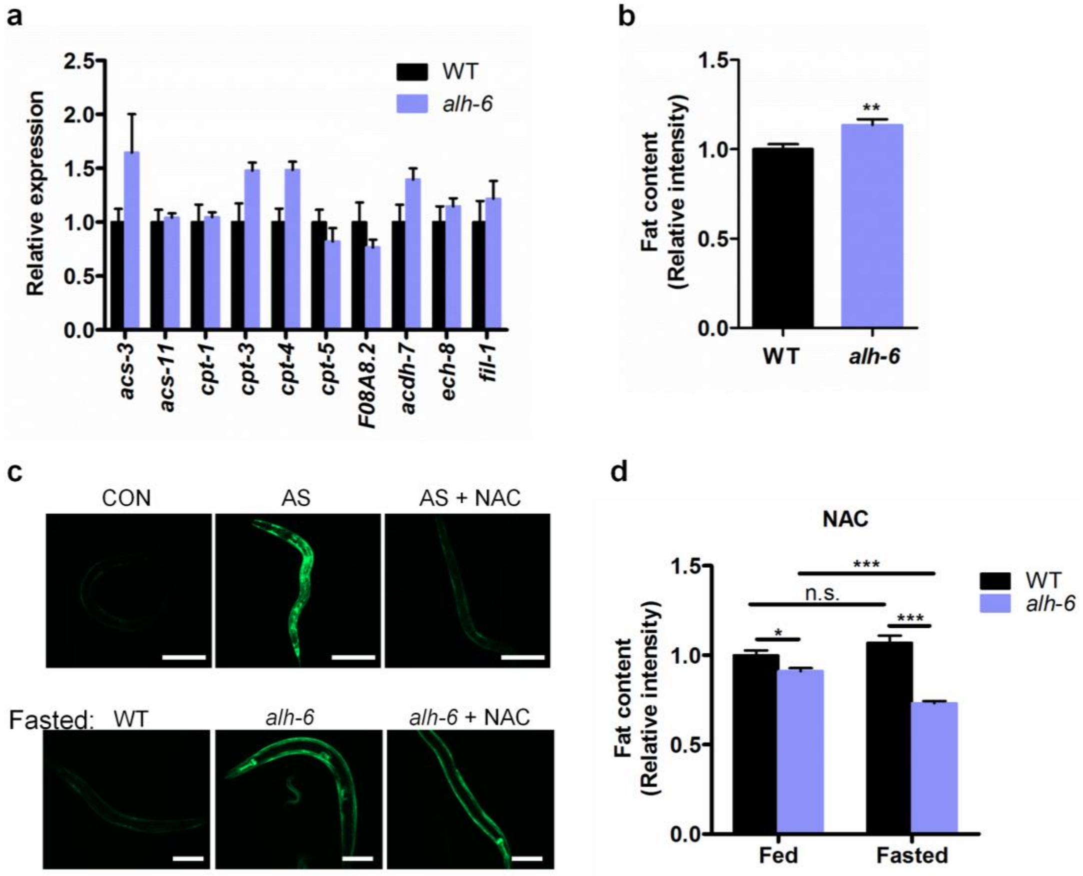

Supplementary Figure 2: ROS is not involved in SKN-1 activation and lipid metabolism in fasted *alh-6* mutants. (a-b), Expression of FAO genes (n = 3) (a) and fat content as measured by Nile Red staining (n = 13 for wild type and n = 16 for *alh-6* mutants) (b) in *alh-6* mutants at day 3 of reproductive period. (c) Antioxidant NAC inhibits arsenite (AS) induced SKN-1 reporter activation but not activation when *alh-6* mutants are fasted. Scale bar: 100um. (d) Fat content of NAC treated worms during fasting as measured by Nile Red staining (n = 8 for fasting *alh-6* mutants, n = 7 for other groups). Data are presented as mean ± SEM. (**p < 0.01, student's t-test, versus wild type controls.)

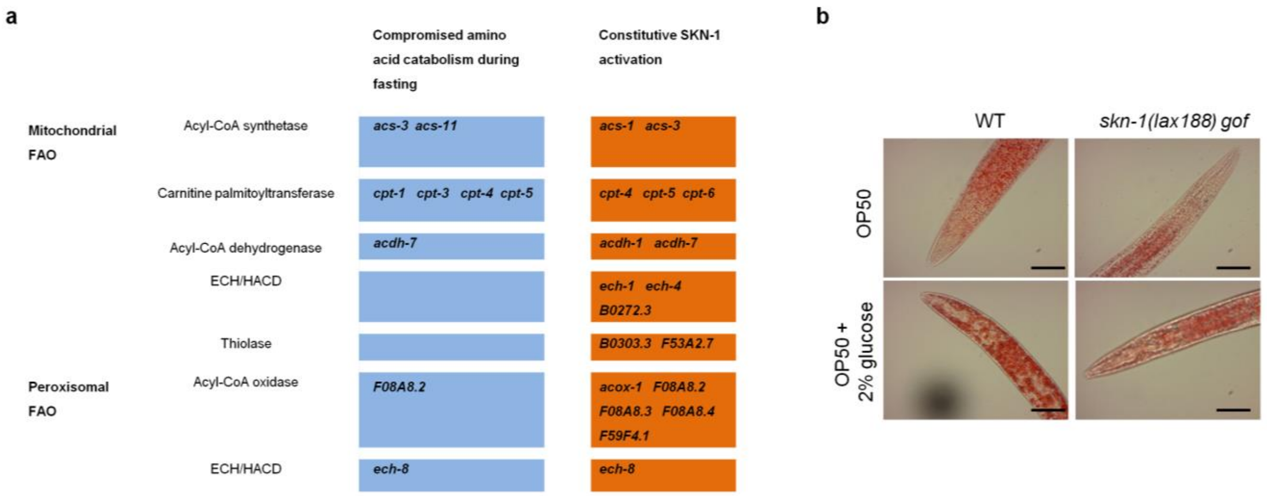

c

Gene	Enzyme	Location	Strand	Sequence
T08B1.6 (*acs-3*)	Acyl-CoA synthetase	-1267	D	ggaaATTTTCATtatc
		-1132	D	agatATTTTCATagaa
		-1822	R	gaaaATTTTCATtaaa
F41C3.3 (*acs-11*)	Acyl-CoA synthetase	-1582	D	cttcTTTTTCATattt
		-51	D	tgaaTTTTTCATttta
		-453	R	tcgaTTTGTCATtctt
		-216	R	acctTATTTCATcaat
Y46G5A.17(*cpt-1*)	Carnitine palmitoyltransferase	-1891	D	gaaaAATATCATaaat
		-1769	R	ggaaATTTTCATtttg
		-947	R	aaatTATATCATatcc
Y48G9A.10(*cpt-3*)	Carnitine palmitoyltransferase	---	---	---
K11D12.4(*cpt-4*)	Carnitine palmitoyltransferase	-1884	D	ttaaAATTTCATtttt
		-1373	D	tattATTATCATtttc
		-660	D	aaaaAATTTCATatta
		-185	D	cagtTATATCATtatc
		-70	D	ccccTATATCATtggg
		-690	R	tggtAATTTCATcaag
T25G12.5	Acyl-CoA dehydrogenase	-1008	D	ggacAATTTCATtttc
		-635	D	tactTTTTTCATtgat
		-39	D	tcgaTTTTTCATagca
F01G10.2(*ech-8*)	enoyl-CoA hydratase	-1167	D	tatcTTTGTCATacac
		-1066	D	tggaAATATCATtgaa
		-209	D	atgaTTTTTCATcgat

Supplementary Figure 3: Lipid metabolism genes and steady state fat levels influenced by SKN-1. (a) Comparison of FAO genes that are deregulated in starved animals with compromised amino acid catabolism or well-fed animals with constitutively activated SKN-1. Genes that are increased by impaired amino acid catabolism during fasting are listed in the blue box; genes upregulated in well-fed constitutively activated SKN-1 mutants are listed in the orange box. (b) Oil Red O staining of worms with indicated genotypes fed OP50 or OP50 supplemented with 2% glucose. Scale bar: 50um. (c) Predicted SKN-1 binding sites WWTDTATC were detected within a 2 kb promoter region of each gene using Regulatory Sequence Analysis Tools (RSAT). D: Sense strand, R: antisense strand.

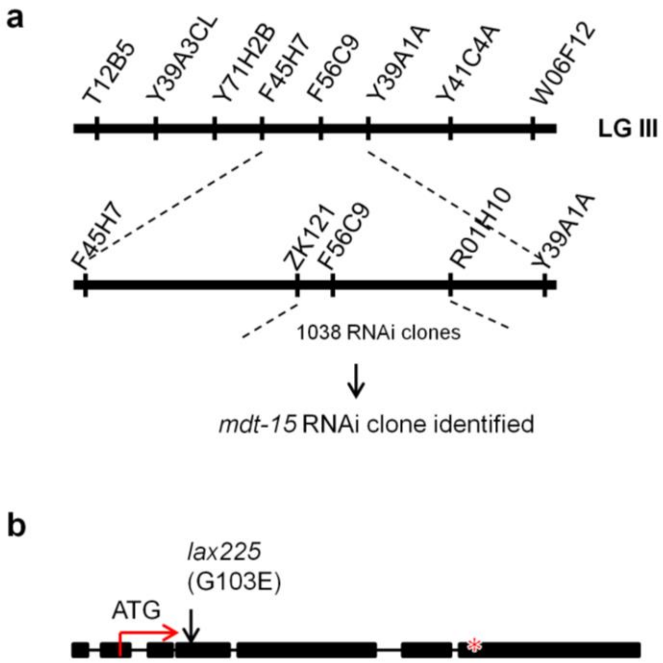

Supplementary Figure 4: Mapping the *lax225* mutation to *mdt-15.* (a) *lax225* is an allele that suppresses the SKN-1 reporter activation in the *skn*-1 gain-of-function mutant background. Through standard SNP mapping, *lax225* was linked to the center of LGIII. Further SNP mapping narrowed the genetic region between ZK121 and R01H10. 1038 RNAi clones covering this region were tested for suppression of the SKN-1 reporter activation in the *skn*-1 gain-of-function mutant background. A single RNAi clone targeting *mdt-15* was identified. (b) Sequencing of *mdt-15* in *lax225* mutants identified a point mutation that causes a Gly to Glu change. Star: stop codon.

Supplementary Table 1: Expression data of all annotated FAO genes in *alh-6* mutant worms after three hours of fasting.

Enzymes	Genes	Fold change (fasted *alh-6*/fasted WT)	P value
Acyl-CoA Synthetase	*acs-2*	0.75	0.133
	acs-3	**1.90**	**0.026**
	acs-11	**1.37**	**0.002**
	acs-13	1.07	0.623
	acs-18	0.89	0.353
	R09E10.4	0.97	0.866
Carnitine Palmitoyl Transferase	***cpt-1***	**2.07**	**0.010**
	cpt-3	**2.09**	**0.043**
	cpt-4	**1.62**	**0.020**
	cpt-5	**1.83**	**0.009**
	cpt-6	1.43	0.058
Acyl-CoA Dehydrogenase	*acdh-1*	0.98	0.937
	acdh-2	0.77	0.116
	acdh-3	1.12	0.445
	acdh-7	**1.43**	**0.048**
	acdh-8	0.98	0.885
	acdh-9	1.29	0.099
	acdh-12	1.17	0.256
Mitochondrial ECH/HACD	*ech-1*	1.02	0.932
	ech-2	0.99	0.933
	ech-4	1.09	0.759
	ech-5	0.78	0.332
	ech-6	1.00	0.976
	ech-7	1.01	0.863
	hacd-1	0.81	0.420
	B0272.3	1.23	0.142
	F54C8.1	1.12	0.400
	T08B2.7	1.17	0.140
Mitochondrial Thiolase	*F53A2.7*	0.90	0.495
	B0303.3	1.18	0.316
Acyl-CoA Oxidase	*acox-1*	1.30	0.053
	F08A8.2	**1.34**	**0.036**
	F08A8.3	1.19	0.354
	F08A8.4	0.95	0.745

	F59F4.1	0.99	0.912
	C48B4.1	0.97	0.625
Peroxisomal ECH/HACD	*ech-3*	1.36	0.064
	ech-8	**1.62**	**0.006**
	ech-9	0.61	0.288
Peroxisomal Thiolase	*T02G5.7*	1.46	0.588

Supplementary Table 2: qPCR primer sequences.

C. elegans Genes	Forward sequences	Reverse sequences
acs-2	AAGGAGATGAGAATGACTGAT	GTTCCGACATGGTGACTA
acs-3	CACGATTCAAGCAACTTC	TTCACTTCCTTATTCTCCATTA
acs-11	GCTTATTGGAATTATGAAGAAG	GGACCTTAGTGATGTGAT
acs-13	ATCAGGCAGAGATCAAGA	GGTTCCATCACAACAAGT
acs-18	GCTTACAATGTCCTATCG	TCCATCTTCTTGAATAATCG
R09E10.4	ATTGATACTGGCGATGAA	AGAGATTGGTTATGTAATGC
cpt-1	TCTATTGTCGTGGAGTCT	GGATTGCGTCGTATTGTA
cpt-3	TACACGGATTCTATGAAGT	TAGTTGTCTGTGATTAGGT
cpt-4	CCAGCACTTCAGGATACT	AGCAGTTGGTCATAGTCTT
cpt-5	GACAGCACAATTCGTAGTA	TCTCCAGCCAACATATCT
cpt-6	CACTTCTACTTCTCATACA	TCACAAGATTCAAGGATT
acdh-1	CTCTGTTCTGATAGTCTT	CCTCTCCTGAATTAGTAA
acdh-2	CAGGAACCATTGCTCAAG	ATTCAGAACTTCAATAGCGTAT
acdh-3	CACAAGTCGTCAATTCTG	TGAGCCAATCCTAACATT
acdh-7	ACCAAGTGTGAGAAGAAG	CGAAGAACCAGTTAGCAT
acdh-8	AATCATCAAGGAGTTCAAT	AAGAGACACCATAAGAGT
acdh-9	TTCTGTCTTATGATTGAGGAT	CTTGTTGGTTGTGAGTTC
acdh-12	AACTCTTGGAATGGTGATG	AACTGCTCTTGGAATGTC
ech-1	CGAATGTAACTATCAATAAGG	ATGGCGGAATAATCAATT
ech-2	GAGTTGAAGGCTATTGAC	ATATTGCGGATGAAGTTC
ech-4	AACGCATTGACATTGGAA	CATTGGCAGTAATCACAGT
ech-5	GTGTGGTGATTCTCAACTC	CTGCTGGCTCATAGTCTT
ech-6	TGGATACTGATAAGTCTGT	CTCGTTATTGGTCATCTC
ech-7	TGTTCTGTTGGCTGATAG	TTTAGGCTGGTTTGGTAG
hacd-1	GCTTGTAGAAGTTGTATC	AATGAATCCAGGAGTATC
B0272.3	TGTGGATAGCAATCAATCTG	CTTCTTCTTGGCAACTCTC
F54C8.1	GATAGTCCTGGATTCATTGT	GCATCTCCTCGTTCATAC
T08B2.7	TGCTCAGGAACTTGCTAA	GTCATAACTGCTTGCGTAG
F53A2.7	AAGTTACCAGACAAGAAG	ATAGTGATTCCAACGATT
B0303.3	GAAGAGAACAAGACGAAT	TAAGAATACTGGAACAACAT
acox-1	CTTCAACAACTACCGTAT	AGCATATAACTTCTCACAT
F08A8.2	ATCTAACCAGCCTGAATG	ATGCCACTTCTCTTGATT
F08A8.3	ACGATACAATGTTCATAC	AATAATCTCCTTCCTTCTA
F08A8.4	CAGCAGCATCGTCTATTC	GTTCAATACCTTCTCCAGTT
F59F4.1	ACTCACTATCCACTCAAG	CGTATCTCATCAGCAATG
C48B4.1	CTCTGATGTTCTTGCTGAT	ATGCTTGTCTTGCTTGAT
ech-3	GAGCACTGGATATGATAC	CCGAGATTCTATTGACAA
ech-8	GAGGAAGGAATCATCCATCAT	GACCACCAGTAGCAACAG
ech-9	GTATTCAACAGGCTTCTTCAT	CCATTCATATCGGCAACAG
T02G5.7	ACTATTCTTGTTGTTGGA	GCATCTTGTTCTTCTCTA
fil-1	GTGTCAACTATTCCTCATT	GGTTCCGTATTCAATAAGAT

**Human Genes**	**Forward sequences**	**Reverse sequences**
Cpt1	AATAAGCAGTCTCTTGATG	CACTTCTGTATCCTTCTTC
Cpt2	TCTACTTCTTAGGTGAGGAA	CGTGATTGGAATCTGATAAC
Acox1	GCAGCCAGATTAGTAGAA	AACAAGGTCAACAGAAGT
Acox2	CACTTGGCTGTTATGATG	GTTCTCCTGAGTATTGGT
Acads	GATTGTGCTGTGAACTAC	CAACTTGAACTGGATGAC
Acadm	CTGGTGCTGTTGGATTAG	ATATTGCTTGGTGCTCTAC
Acadl	TAGTATTCATTCAGGTATTGTC	GCTCTGTCATTGCTATTG
Ech1	TAGAGTGCTTCAACAAGA	ATGTCCATCAGGTCAATA
Hadha	CCGTCCTTATCTCATCAA	AACTATTCTCTGTGCTTCT
Hadhb	ACACTCACACTAGGCAATG	AACACTGGCAAGGCTTAA
Nrf2	ACACGGTCCACAGCTCATC	TTGACATACTTTGGAGGCAAGA
Gclc	CAAGGACGTTCTCAAGTGGG	AGAGAAGGGGGAAAGGACAA
Gclm	CTGTGTGATGCCACCAGATT	TGAAGCAAGTTTCCAAGAAGC
Nqo1	GGACTGCACCAGAGCCAT	CGGCTTTGAAGAAGAAAGGA

Supplementary Table 3: Starvation survival data.

	Number of surviving animals[1] (% of day 0)			
**OP50:**				
**Days**	**WT**	***alh-6 (lax105)***		
0	157 (100%)	206 (100%)		
2	159 (100%)	212 (100%)		
4	160 (100%)	204 (99%)		
6	155 (99%)	167 (81%)		
8	138 (88%)	97 (47%)		
10	90 (57%)	17 (8%)		
12	70 (45%)	2 (1%)		
14	31 (20%)	0 (0%)		
16	17 (11%)			
18	5 (3%)			
20	0 (0 %)			
**HT115:**				
**Days**	**WT**	***alh-6 (lax105)***		
0	141 (100%)	108 (100%)		
3	128 (91%)	91 (84%)		
6	101 (72%)	47 (44%)		
9	84 (60%)	11 (10%)		
12	32 (23%)	0 (0%)		
15	9 (6%)			
18	0 (0%)			
**OP50:**				
**Days**	***skn-1/nTi***	***alh-6; skn-1/nTi***	***skn-1***	***alh-6; skn-1***
0	204 (100%)	210 (100%)	110 (100%)	100 (100%)
2	220 (100%)	198 (94%)	112 (100%)	100 (100%)
4	207 (100%)	197 (94%)	108 (98%)	102 (100%)
6	199 (98%)	192 (91%)	104 (95%)	98 (98%)
8	198 (97%)	163 (78%)	103 (94%)	77 (77%)
10	168 (82%)	111 (53%)	100 (91%)	75 (75%)
12	166 (81%)	56 (27%)	93 (85%)	50 (50%)
14	144 (71%)	20 (10%)	69 (63%)	19 (19%)
16	134 (66%)	5 (2%)	52 (47%)	12 (12%)
18	107 (52%)	2 (1%)	32 (29%)	2 (2%)
20	51 (25%)	0 (0%)	22 (20%)	0 (0%)
22	16 (8%)		6 (5%)	
24	0 (0%)		0 (0%)	

1. Data represent the average from two biological replicates from each condition.
